# 1-Benzoyl-5-phenyl-2-(propan-2-yl)-1,2,3,4-tetra­hydro­pyrimidin-4-one

**DOI:** 10.1107/S1600536809036356

**Published:** 2009-09-16

**Authors:** Ignez Caracelli, Julio Zukerman-Schpector, Mônica F. Z. J. Amaral, Hélio A. Stefani, Edward R. T. Tiekink

**Affiliations:** a BioMat-Physics Department, Universidade Estadual Paulista Júlio de Mesquita Filho, UNESP, 17033-360 Bauru, SP, Brazil; b Department of Chemistry, Universidade Federal de São Carlos, 13565-905 São Carlos, SP, Brazil; c Departamento de Farmácia, Faculdade de Ciências Farmacêuticas, Universidade de São Paulo, São Paulo, SP, Brazil

## Abstract

The tetra­hydro­pyrimidinone ring in the title compound, C_20_H_20_N_2_O_2_, is in a half-boat conformation with the N—C—N C atom 0.580 (2) Å out of the plane defined by the remaining five atoms. In the crystal structure, mol­ecules are connected into centrosymmetric dimers *via* N—H⋯O inter­actions. The dimeric aggregates are linked into supra­molecular chains along the *a* axis *via* C—H⋯π inter­actions.

## Related literature

For background to the use of potassium organotrifluoro­borate salts in organic synthesis, see: Caracelli *et al.* (2007 ▶); Stefani *et al.* (2007 ▶); Vieira *et al.* (2008 ▶). For a related structure, see: Vega-Teijido *et al.* (2007 ▶). For conformational analysis, see: Cremer & Pople (1975 ▶); Iulek & Zukerman-Schpector (1997 ▶).
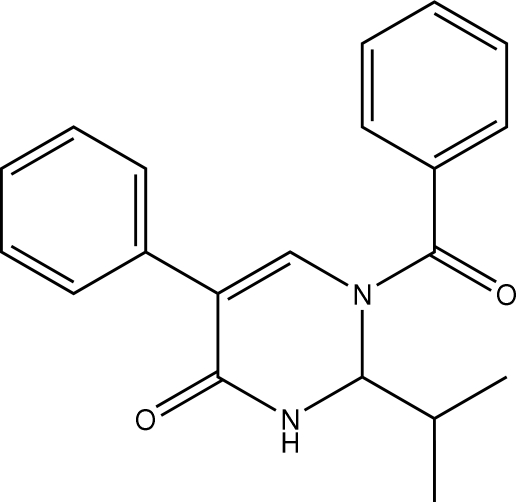


## Experimental

### 

#### Crystal data

C_20_H_20_N_2_O_2_
*M* *_r_* = 320.38
Monoclinic, 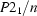

*a* = 9.346 (4) Å
*b* = 8.001 (3) Å
*c* = 22.528 (9) Å
β = 96.843 (9)°
*V* = 1672.6 (12) Å^3^
*Z* = 4
Mo *K*α radiation
μ = 0.08 mm^−1^
*T* = 98 K
0.35 × 0.22 × 0.10 mm

#### Data collection

Rigaku AFC12/SATURN724 diffractometer
Absorption correction: multi-scan *ABSCOR* (Higashi, 1995 ▶) *T*_min_ = 0.811, *T*_max_ = 1
5658 measured reflections
3094 independent reflections
2636 reflections with *I* > 2σ(*I*)
*R*_int_ = 0.035

#### Refinement

*R*[*F* ^2^ > 2σ(*F* ^2^)] = 0.049
*wR*(*F* ^2^) = 0.126
*S* = 1.09
3094 reflections
213 parameters
H-atom parameters constrained
Δρ_max_ = 0.22 e Å^−3^
Δρ_min_ = −0.20 e Å^−3^

### 

Data collection: *CrystalClear* (Rigaku, 2005 ▶); cell refinement: *CrystalClear*; data reduction: *CrystalClear*; program(s) used to solve structure: *SIR97* (Altomare *et al.*, 1999 ▶); program(s) used to refine structure: *SHELXL97* (Sheldrick, 2008 ▶); molecular graphics: *ORTEP-3* (Farrugia, 1997 ▶) and *DIAMOND* (Brandenburg, 2006 ▶); software used to prepare material for publication: *WinGX* (Farrugia, 1999 ▶).

## Supplementary Material

Crystal structure: contains datablocks global, I. DOI: 10.1107/S1600536809036356/ng2639sup1.cif

Structure factors: contains datablocks I. DOI: 10.1107/S1600536809036356/ng2639Isup2.hkl

Additional supplementary materials:  crystallographic information; 3D view; checkCIF report

## Figures and Tables

**Table 1:** Hydrogen-bond geometry (Å, °)

*D*—H⋯*A*	*D*—H	H⋯*A*	*D*⋯*A*	*D*—H⋯*A*
N3—H1*N*3⋯O1^i^	0.93	1.90	2.827 (2)	174
C9—H9⋯*Cg*^ii^	0.93	2.82	3.632 (2)	147

Symmetry codes: (i) 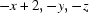; (ii) 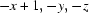. *Cg* is the centroid of the C14–C19 ring.

**Fig. 1.**
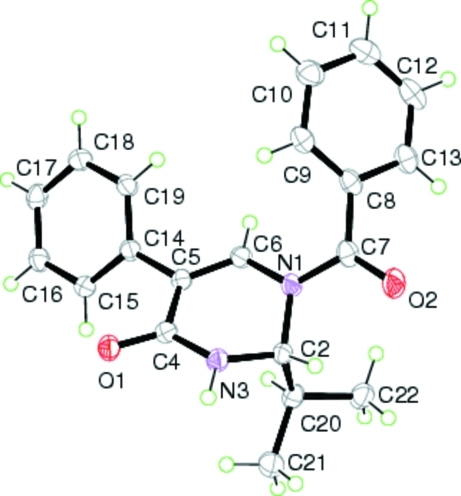
The molecular structure of (I) showing atom labelling scheme and displacement ellipsoids at the 50% probability level (arbitrary spheres for the H atoms).

**Fig. 2.**
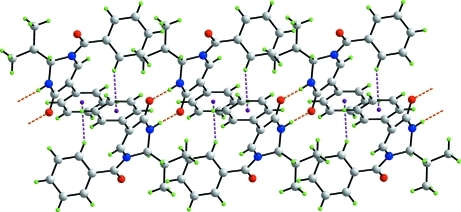
Supramolecular chain in (I) comprising centrosymmetric dimers mediated by {O═C—N—H···}2 synthons (hydrogen bonds shown as orange dashed lines) linked by C—H···π interactions (shown as purple dashed lines).

## supplementary crystallographic information

### Comment

As part of our on-going research interest efforts exploring the chemistry of potassium organotrifluoroborate salts, including their potential use as intermediates in organic synthesis (Caracelli *et al.*, 2007; Stefani *et al.*, 2007; Vieira *et al.* 2008), we have started to use the Suzuki–Miyaura cross-coupling reaction as a new tool to synthesize β-amino acids. Herein, the crystal structure of (I) is described.

The molecular structure of (I), Fig. 1, shows the tetrahydropyrimidinone ring to adopt a half-boat conformation with the C2 atom being displaced 0.580 (2) Å out of the plane defined by the remaining five atoms. The ring-puckering parameters are q_2_ = 0.374 (2) Å, q_3_ = 0.187 (1) Å, Q = 0.418 (2) Å, and φ_2_ = 54.0 (2)° (Cremer & Pople, 1975; Iulek & Zukerman-Schpector, 1997). The dihedral angle between the aryl rings is 55.55 (8)°. The deviation of the torsion angle C4—C5—C14—C15 from the ideal value of 60°, which would indicate bisection of the dihydripyrimidinone ring by the plane of the phenyl ring, is of 15.7°.

In the crystal packing, centrosymmetric dimers are formed being consolidated by eight-membered {O═C—N—H···}_2_ synthons, Table 1. The carbonyl-O2 atom is involved in an intramolecular C—H···O contact with the C—H2 atom (2.34 Å) and does not particiate in a significant intermolecular contact. The aggregates thus formed are linked into supramolecular chains, Fig. 2, aligned along the *a* axis *via* C—H···π interactions: C9—H9···*Cg*(C14–C19)^i^ = 2.82 Å, C9···*Cg*(C14–C19)^i^ = 3.632 (2) Å with an angle of 147° at H9; symmetry operation i: 1 - *x*, -*y*, -*z*.

### Experimental

A 50 ml flask under N_2_ atmosphere was charged with potassium phenyltrifluoroborate (1.2 mmol), (*S*)-5-iodopyrimidinone 3 (1.0 mmol, 370 mg), Pd(OAc)_2_ (9 mol%, 20.02 mg), K_2_CO_3_ (2 mmol, 276 mg), and 16 ml of degassed dioxane/H_2_O (3/1). The reaction mixture was refluxed at 383 K and the reaction followed by TLC and GC. After completion, the reaction mixture was cooled and then extracted with ethyl acetate (3 × 50 ml). The organic layers were combined, dried (MgSO_4_), and the solvent removed under vacuum to give a viscous oil. The oil was purified *via* column chromatography using a mixture of ethyl acetate/hexane (1:1) as the eluent. Single crystals of (I) were obtained by slow evaporation from ethyl acetate.

### Refinement

The H atoms were positioned with idealized geometry using a riding model with N—H = 0.93 Å and C—H = 0.93–0.98 Å, and with *U*_iso_ set to 1.2 times (1.5 for methyl) *U*_eq_(parent atom).

### Crystal data

**Table:**

C_20_H_20_N_2_O_2_	*F*(000) = 680
*M**_r_* = 320.38	*D*_x_ = 1.272 Mg m^−^^3^
Monoclinic, *P*2_1_/*n*	Mo *K*α radiation, λ = 0.71073 Å
Hall symbol: -P 2yn	Cell parameters from 6484 reflections
*a* = 9.346 (4) Å	θ = 2.5–40.2°
*b* = 8.001 (3) Å	µ = 0.08 mm^−^^1^
*c* = 22.528 (9) Å	*T* = 98 K
β = 96.843 (9)°	Prism, colourless
*V* = 1672.6 (12) Å^3^	0.35 × 0.22 × 0.10 mm
*Z* = 4	

### Data collection

**Table:**

Rigaku AFC12/SATURN724 diffractometer	3094 independent reflections
Radiation source: fine-focus sealed tube	2636 reflections with *I* > 2σ(*I*)
graphite	*R*_int_ = 0.035
ω scans	θ_max_ = 25.5°, θ_min_ = 2.5°
Absorption correction: multi-scan *ABSCOR* (Higashi, 1995)	*h* = −11→8
*T*_min_ = 0.811, *T*_max_ = 1	*k* = −6→9
5658 measured reflections	*l* = −26→27

### Refinement

**Table:**

Refinement on *F*^2^	Primary atom site location: structure-invariant direct methods
Least-squares matrix: full	Secondary atom site location: difference Fourier map
*R*[*F*^2^ > 2σ(*F*^2^)] = 0.049	Hydrogen site location: inferred from neighbouring sites
*wR*(*F*^2^) = 0.126	H-atom parameters constrained
*S* = 1.09	*w* = 1/[σ^2^(*F*_o_^2^) + (0.0541*P*)^2^ + 0.6346*P*] where *P* = (*F*_o_^2^ + 2*F*_c_^2^)/3
3094 reflections	(Δ/σ)_max_ < 0.001
213 parameters	Δρ_max_ = 0.22 e Å^−^^3^
0 restraints	Δρ_min_ = −0.20 e Å^−^^3^

### Special details

**Table:**

Geometry. All s.u.'s (except the s.u. in the dihedral angle between two l.s. planes) are estimated using the full covariance matrix. The cell s.u.'s are taken into account individually in the estimation of s.u.'s in distances, angles and torsion angles; correlations between s.u.'s in cell parameters are only used when they are defined by crystal symmetry. An approximate (isotropic) treatment of cell s.u.'s is used for estimating s.u.'s involving l.s. planes.
Refinement. Refinement of *F*^2^ against ALL reflections. The weighted *R*-factor *wR* and goodness of fit *S* are based on *F*^2^, conventional *R*-factors *R* are based on *F*, with *F* set to zero for negative *F*^2^. The threshold expression of *F*^2^ > σ(*F*^2^) is used only for calculating *R*-factors(gt) *etc*. and is not relevant to the choice of reflections for refinement. *R*-factors based on *F*^2^ are statistically about twice as large as those based on *F*, and *R*- factors based on ALL data will be even larger.

### Fractional atomic coordinates and isotropic or equivalent isotropic displacement parameters (Å^2^)

**Table:**

	*x*	*y*	*z*	*U*_iso_*/*U*_eq_	
C2	0.91894 (17)	0.2343 (2)	0.10191 (7)	0.0228 (4)	
H2	0.9791	0.1868	0.1364	0.027*	
C4	0.81099 (17)	0.1247 (2)	0.00497 (7)	0.0227 (4)	
C5	0.68065 (16)	0.2234 (2)	0.01348 (7)	0.0215 (4)	
C6	0.66388 (17)	0.2745 (2)	0.06922 (7)	0.0225 (4)	
H6	0.5782	0.3264	0.0759	0.027*	
C7	0.74672 (18)	0.2425 (2)	0.17669 (7)	0.0250 (4)	
C8	0.59340 (18)	0.2378 (2)	0.19000 (7)	0.0249 (4)	
C9	0.49283 (19)	0.1281 (2)	0.16115 (8)	0.0289 (4)	
H9	0.5177	0.0606	0.1304	0.035*	
C10	0.3547 (2)	0.1194 (3)	0.17838 (8)	0.0346 (4)	
H10	0.2877	0.0448	0.1596	0.041*	
C11	0.3174 (2)	0.2217 (3)	0.22339 (9)	0.0362 (5)	
H11	0.2248	0.2171	0.2345	0.043*	
C12	0.4177 (2)	0.3315 (2)	0.25215 (8)	0.0346 (4)	
H12	0.3917	0.4005	0.2823	0.041*	
C13	0.5563 (2)	0.3389 (2)	0.23625 (8)	0.0296 (4)	
H13	0.6239	0.4107	0.2562	0.036*	
C14	0.56628 (17)	0.2461 (2)	−0.03774 (7)	0.0219 (4)	
C15	0.60156 (17)	0.2860 (2)	−0.09467 (7)	0.0235 (4)	
H15	0.6977	0.2993	−0.1005	0.028*	
C16	0.49504 (18)	0.3061 (2)	−0.14248 (7)	0.0264 (4)	
H16	0.5201	0.3327	−0.1801	0.032*	
C17	0.35113 (18)	0.2865 (2)	−0.13443 (8)	0.0279 (4)	
H17	0.2796	0.3000	−0.1665	0.033*	
C18	0.31473 (18)	0.2469 (2)	−0.07842 (8)	0.0285 (4)	
H18	0.2184	0.2339	−0.0729	0.034*	
C19	0.42147 (17)	0.2262 (2)	−0.03020 (7)	0.0245 (4)	
H19	0.3958	0.1990	0.0073	0.029*	
C20	0.98292 (17)	0.4034 (2)	0.08670 (7)	0.0260 (4)	
H20	0.9135	0.4597	0.0573	0.031*	
C21	1.12258 (19)	0.3783 (2)	0.05890 (8)	0.0323 (4)	
H21A	1.1605	0.4851	0.0492	0.048*	
H21B	1.1035	0.3125	0.0232	0.048*	
H21C	1.1916	0.3215	0.0868	0.048*	
C22	1.01001 (18)	0.5154 (2)	0.14175 (8)	0.0318 (4)	
H22A	0.9239	0.5223	0.1610	0.048*	
H22B	1.0367	0.6252	0.1298	0.048*	
H22C	1.0865	0.4692	0.1691	0.048*	
N1	0.77103 (14)	0.25198 (17)	0.11728 (6)	0.0226 (3)	
N3	0.91274 (14)	0.11564 (18)	0.05278 (6)	0.0232 (3)	
H1N3	0.9978	0.0622	0.0463	0.028*	
O1	0.82249 (12)	0.04539 (16)	−0.04171 (5)	0.0292 (3)	
O2	0.84733 (13)	0.23635 (17)	0.21657 (5)	0.0335 (3)	

### Atomic displacement parameters (Å^2^)

**Table:**

	*U*^11^	*U*^22^	*U*^33^	*U*^12^	*U*^13^	*U*^23^
C2	0.0202 (8)	0.0296 (9)	0.0179 (8)	0.0028 (7)	0.0000 (6)	−0.0023 (7)
C4	0.0207 (8)	0.0246 (8)	0.0227 (8)	−0.0016 (7)	0.0022 (6)	−0.0002 (7)
C5	0.0201 (8)	0.0254 (8)	0.0191 (8)	−0.0008 (7)	0.0022 (6)	0.0008 (7)
C6	0.0198 (7)	0.0273 (8)	0.0203 (8)	0.0023 (7)	0.0018 (6)	0.0029 (7)
C7	0.0308 (9)	0.0247 (9)	0.0192 (8)	0.0059 (7)	0.0015 (7)	0.0028 (7)
C8	0.0307 (9)	0.0265 (9)	0.0180 (8)	0.0051 (7)	0.0047 (6)	0.0049 (7)
C9	0.0337 (9)	0.0316 (9)	0.0220 (8)	0.0023 (8)	0.0061 (7)	0.0021 (7)
C10	0.0345 (10)	0.0382 (11)	0.0318 (10)	−0.0026 (8)	0.0072 (7)	0.0062 (8)
C11	0.0342 (10)	0.0420 (11)	0.0346 (10)	0.0079 (9)	0.0129 (8)	0.0104 (9)
C12	0.0454 (11)	0.0349 (10)	0.0257 (9)	0.0146 (9)	0.0137 (8)	0.0055 (8)
C13	0.0383 (10)	0.0281 (9)	0.0226 (8)	0.0056 (8)	0.0041 (7)	0.0020 (7)
C14	0.0223 (8)	0.0227 (8)	0.0206 (8)	0.0007 (7)	0.0026 (6)	−0.0009 (6)
C15	0.0220 (8)	0.0266 (8)	0.0220 (8)	−0.0008 (7)	0.0030 (6)	−0.0003 (7)
C16	0.0306 (9)	0.0292 (9)	0.0191 (8)	−0.0001 (7)	0.0024 (7)	0.0012 (7)
C17	0.0272 (8)	0.0317 (9)	0.0228 (8)	0.0052 (8)	−0.0049 (6)	−0.0012 (7)
C18	0.0199 (8)	0.0370 (10)	0.0283 (9)	0.0023 (7)	0.0012 (7)	−0.0021 (8)
C19	0.0232 (8)	0.0311 (9)	0.0194 (8)	0.0012 (7)	0.0037 (6)	0.0007 (7)
C20	0.0243 (8)	0.0282 (9)	0.0242 (8)	0.0011 (7)	−0.0026 (6)	−0.0001 (7)
C21	0.0334 (9)	0.0343 (10)	0.0297 (9)	−0.0054 (8)	0.0060 (7)	−0.0005 (8)
C22	0.0281 (9)	0.0325 (10)	0.0338 (10)	0.0004 (8)	−0.0006 (7)	−0.0090 (8)
N1	0.0212 (7)	0.0292 (8)	0.0169 (7)	0.0030 (6)	0.0008 (5)	0.0012 (6)
N3	0.0196 (6)	0.0270 (7)	0.0223 (7)	0.0034 (6)	0.0003 (5)	−0.0030 (6)
O1	0.0247 (6)	0.0379 (7)	0.0243 (6)	0.0044 (5)	0.0001 (4)	−0.0091 (5)
O2	0.0325 (7)	0.0468 (8)	0.0197 (6)	0.0053 (6)	−0.0031 (5)	0.0007 (6)

### Geometric parameters (Å, °)

**Table:**

C2—N3	1.454 (2)	C12—H12	0.9300
C2—N1	1.471 (2)	C13—H13	0.9300
C2—C20	1.534 (2)	C14—C19	1.393 (2)
C2—H2	0.9800	C14—C15	1.399 (2)
C4—O1	1.244 (2)	C15—C16	1.386 (2)
C4—N3	1.351 (2)	C15—H15	0.9300
C4—C5	1.483 (2)	C16—C17	1.387 (2)
C5—C6	1.347 (2)	C16—H16	0.9300
C5—C14	1.488 (2)	C17—C18	1.382 (3)
C6—N1	1.396 (2)	C17—H17	0.9300
C6—H6	0.9300	C18—C19	1.394 (2)
C7—O2	1.222 (2)	C18—H18	0.9300
C7—N1	1.386 (2)	C19—H19	0.9300
C7—C8	1.499 (2)	C20—C22	1.526 (2)
C8—C9	1.389 (3)	C20—C21	1.527 (2)
C8—C13	1.395 (2)	C20—H20	0.9800
C9—C10	1.394 (2)	C21—H21A	0.9600
C9—H9	0.9300	C21—H21B	0.9600
C10—C11	1.380 (3)	C21—H21C	0.9600
C10—H10	0.9300	C22—H22A	0.9600
C11—C12	1.387 (3)	C22—H22B	0.9600
C11—H11	0.9300	C22—H22C	0.9600
C12—C13	1.386 (3)	N3—H1N3	0.9300
			
N3—C2—N1	106.75 (12)	C16—C15—C14	120.86 (15)
N3—C2—C20	112.78 (14)	C16—C15—H15	119.6
N1—C2—C20	111.71 (13)	C14—C15—H15	119.6
N3—C2—H2	108.5	C15—C16—C17	120.20 (15)
N1—C2—H2	108.5	C15—C16—H16	119.9
C20—C2—H2	108.5	C17—C16—H16	119.9
O1—C4—N3	121.55 (15)	C18—C17—C16	119.55 (15)
O1—C4—C5	122.39 (14)	C18—C17—H17	120.2
N3—C4—C5	115.88 (14)	C16—C17—H17	120.2
C6—C5—C4	118.07 (15)	C17—C18—C19	120.48 (16)
C6—C5—C14	122.20 (15)	C17—C18—H18	119.8
C4—C5—C14	119.36 (14)	C19—C18—H18	119.8
C5—C6—N1	122.08 (15)	C14—C19—C18	120.44 (16)
C5—C6—H6	119.0	C14—C19—H19	119.8
N1—C6—H6	119.0	C18—C19—H19	119.8
O2—C7—N1	120.79 (16)	C22—C20—C21	110.07 (14)
O2—C7—C8	121.49 (15)	C22—C20—C2	111.56 (15)
N1—C7—C8	117.71 (14)	C21—C20—C2	110.53 (14)
C9—C8—C13	120.04 (16)	C22—C20—H20	108.2
C9—C8—C7	122.16 (15)	C21—C20—H20	108.2
C13—C8—C7	117.61 (16)	C2—C20—H20	108.2
C8—C9—C10	119.94 (17)	C20—C21—H21A	109.5
C8—C9—H9	120.0	C20—C21—H21B	109.5
C10—C9—H9	120.0	H21A—C21—H21B	109.5
C11—C10—C9	119.90 (18)	C20—C21—H21C	109.5
C11—C10—H10	120.0	H21A—C21—H21C	109.5
C9—C10—H10	120.0	H21B—C21—H21C	109.5
C10—C11—C12	120.21 (17)	C20—C22—H22A	109.5
C10—C11—H11	119.9	C20—C22—H22B	109.5
C12—C11—H11	119.9	H22A—C22—H22B	109.5
C13—C12—C11	120.43 (17)	C20—C22—H22C	109.5
C13—C12—H12	119.8	H22A—C22—H22C	109.5
C11—C12—H12	119.8	H22B—C22—H22C	109.5
C12—C13—C8	119.46 (18)	C7—N1—C6	124.81 (14)
C12—C13—H13	120.3	C7—N1—C2	119.25 (13)
C8—C13—H13	120.3	C6—N1—C2	115.93 (13)
C19—C14—C15	118.46 (15)	C4—N3—C2	122.15 (14)
C19—C14—C5	120.67 (15)	C4—N3—H1N3	115.7
C15—C14—C5	120.87 (14)	C2—N3—H1N3	117.6
			
O1—C4—C5—C6	165.73 (16)	C14—C15—C16—C17	−0.1 (3)
N3—C4—C5—C6	−9.5 (2)	C15—C16—C17—C18	0.0 (3)
O1—C4—C5—C14	−7.5 (2)	C16—C17—C18—C19	−0.1 (3)
N3—C4—C5—C14	177.25 (14)	C15—C14—C19—C18	−0.4 (3)
C4—C5—C6—N1	6.5 (2)	C5—C14—C19—C18	−179.68 (16)
C14—C5—C6—N1	179.57 (15)	C17—C18—C19—C14	0.3 (3)
O2—C7—C8—C9	−129.50 (19)	N3—C2—C20—C22	−171.05 (13)
N1—C7—C8—C9	49.6 (2)	N1—C2—C20—C22	68.70 (17)
O2—C7—C8—C13	45.5 (2)	N3—C2—C20—C21	−48.23 (18)
N1—C7—C8—C13	−135.33 (17)	N1—C2—C20—C21	−168.48 (13)
C13—C8—C9—C10	0.1 (3)	O2—C7—N1—C6	−173.99 (16)
C7—C8—C9—C10	175.01 (16)	C8—C7—N1—C6	6.9 (2)
C8—C9—C10—C11	1.1 (3)	O2—C7—N1—C2	7.3 (2)
C9—C10—C11—C12	−1.0 (3)	C8—C7—N1—C2	−171.89 (14)
C10—C11—C12—C13	−0.3 (3)	C5—C6—N1—C7	−155.06 (17)
C11—C12—C13—C8	1.5 (3)	C5—C6—N1—C2	23.7 (2)
C9—C8—C13—C12	−1.4 (3)	N3—C2—N1—C7	132.30 (15)
C7—C8—C13—C12	−176.52 (15)	C20—C2—N1—C7	−104.00 (17)
C6—C5—C14—C19	−38.0 (2)	N3—C2—N1—C6	−46.57 (18)
C4—C5—C14—C19	134.97 (17)	C20—C2—N1—C6	77.13 (17)
C6—C5—C14—C15	142.74 (18)	O1—C4—N3—C2	165.52 (15)
C4—C5—C14—C15	−44.3 (2)	C5—C4—N3—C2	−19.2 (2)
C19—C14—C15—C16	0.3 (3)	N1—C2—N3—C4	46.0 (2)
C5—C14—C15—C16	179.55 (15)	C20—C2—N3—C4	−77.04 (19)

### Hydrogen-bond geometry (Å, °)

**Table:**

*D*—H···*A*	*D*—H	H···*A*	*D*···*A*	*D*—H···*A*
N3—H1N3···O1^i^	0.93	1.90	2.827 (2)	174
C9—H9···Cg^ii^	0.93	2.82	3.632 (2)	147

Symmetry codes: (i) −*x*+2, −*y*, −*z*; (ii) −*x*+1, −*y*, −*z*.

## References

[bb1] Altomare, A., Burla, M. C., Camalli, M., Cascarano, G. L., Giacovazzo, C., Guagliardi, A., Moliterni, A. G. G., Polidori, G. & Spagna, R. (1999). *J. Appl. Cryst.* **32**, 115–119.

[bb2] Brandenburg, K. (2006). *DIAMOND* Crystal Impact GbR, Bonn, Germany.

[bb3] Caracelli, I., Stefani, H. A., Vieira, A. S., Machado, M. M. P. & Zukerman-Schpector, J. (2007). *Z. Kristallogr. New Cryst. Struct.* **222**, 345–346.

[bb4] Cremer, D. & Pople, J. A. (1975). *J. Am. Chem. Soc.* **97**, 1354–1358.

[bb5] Farrugia, L. J. (1997). *J. Appl. Cryst.* **30**, 565.

[bb6] Farrugia, L. J. (1999). *J. Appl. Cryst.* **32**, 837–838.

[bb7] Higashi, T. (1995). *ABSCOR* Rigaku Corporation, Tokyo, Japan.

[bb8] Iulek, J. & Zukerman-Schpector, J. (1997). *Quim. Nova*, **20**, 433–434.

[bb9] Rigaku (2005). *CrystalClear* Rigaku/MSC Inc., The Woodlands, Texas, USA.

[bb10] Sheldrick, G. M. (2008). *Acta Cryst.* A**64**, 112–122. 10.1107/S0108767307043930 18156677

[bb11] Stefani, H. A., Cella, R. & Vieira, A. S. (2007). *Tetrahedron*, **63**, 3623–3658.

[bb12] Vega-Teijido, M., Zukerman-Schpector, J., Nunes, F. M., Gatti, P. M., Stefani, H. A. & Caracelli, I. (2007). *Z. Kristallogr.* **222**, 705–712.

[bb13] Vieira, A. S., Fiorante, P. F., Zukerman-Schpector, J., Alves, D., Botteselle, G. V. & Stefani, H. A. (2008). *Tetrahedron*, **64**, 7234–7241.

